# Thin Film and Nanostructured Pd-Based Materials for Optical H_2_ Sensors: A Review

**DOI:** 10.3390/nano11113100

**Published:** 2021-11-17

**Authors:** Andreas Sousanis, George Biskos

**Affiliations:** 1 Climate and Atmosphere Research Centre, The Cyprus Institute, Nicosia 2121, Cyprus; a.sousanis@cyi.ac.cy; 2 Faculty of Civil Engineering and Geosciences, Delft University of Technology, 2628 CN Delft, The Netherlands

**Keywords:** Pd-based H_2_ sensors, optical H_2_ sensors, thin film-based sensors, nanostructured sensors, nanoparticle-based sensors

## Abstract

In this review paper, we provide an overview of state-of-the-art Pd-based materials for optical H_2_ sensors. The first part of the manuscript introduces the operating principles, providing background information on the thermodynamics and the primary mechanisms of optical detection. Optical H_2_ sensors using thin films (i.e., films without any nanostructuring) are discussed first, followed by those employing nanostructured materials based on aggregated or isolated nanoparticles (ANPs and INPs, respectively), as well as complex nanostructured (CN) architectures. The different material types are discussed on the basis of the properties they can attribute to the resulting sensors, including their limit of detection, sensitivity, and response time. Limitations induced by cracking and the hysteresis effect, which reduce the repeatability and reliability of the sensors, as well as by CO poisoning that deteriorates their performance in the long run, are also discussed together with an overview of manufacturing approaches (e.g., tailoring the composition and/or applying functionalizing coatings) for addressing these issues.

## 1. Introduction

Hydrogen (H_2_) is a clean energy carrier that provides a promising alternative to fossil fuels as its only by-product when consumed is water [1,2,3,4]. Despite the unique eco-friendly nature and high energy density, the high flammability over a wide concentration range (4–75%) and low ignition energy (0.0017 mJ) of H_2_ are important concerns for its safe handling and use [5,6]. This, in turn, raises the need to develop reliable sensors capable of measuring H_2_ over a wide range of concentrations (from a few tens of ppb to a few percent) under different environmental conditions.

To guide sensor manufacturing that can fulfil the needs of the H_2_ industry, the US Department of Energy has set a number of target specifications. For example, the sensors should be able to measure H_2_ at concentrations ranging from 0.01 to 4% in air. In addition, they should have fast response (in the range of 1 s or shorter) and recovery times, while the uncertainty of the recorded concentration needs to be less than 5–10%. Another important specification is that the performance of the sensors must be stable over wide temperature (e.g., from −30 to 80 °C) and humidity ranges.

Based on their operating principle, the most common types of H_2_ sensors can be classified as (1) electrical or (2) optical [7,8]. Electrical H_2_ sensors available in the market have detection limits down to a few hundreds of ppm and response times in the order of tens of seconds. Recent efforts have yielded electrical H_2_ sensors that exhibit superior performance compared to their commercially available counterparts, with reported limit of detection (LOD) down to a couple of tens of ppb, and response times of less than one second [9,10,11,12,13,14].

Electrical sensors [15] rely on changes in the resistance (or conductance) of their sensing elements that are induced by concentration variabilities of H_2_ in the gas they are exposed to. The most common electrical sensors employ metal oxide semiconductors (e.g., ZnO, NiO, and TiO_2_) as sensing elements [16,17]. The resistance of these materials is sensitive to the depletion of electrons on their surface that is caused by the adsorption of target gas molecules [18], providing a measurable variable related to the concentration of specific gases. The thickness of the electron depletion region increases as a function of the number of gaseous H_2_ molecules adsorbing on the surface of the metal oxide (which is proportional to the concentration of H_2_ in the vicinity of the sensing material), and returns to that corresponding to the initial/nominal resistance of the material when H_2_ is removed. Two substantial limitations of sensors that employ metal oxide semiconductors are (1) the limited selectivity, and (2) the high operating temperature (which can typically vary between 180 and 500 °C) needed for optimal performance [19,20]. These limitations can be addressed by introducing catalytic nanoparticles on the sensing material as has been demonstrated by a number of studies [21,22,23].

Apart from MOS-based hydrogen sensors, other categories of sensors for measuring the concentration of H_2_ in gases can be classified into three main categories: conductometric, amperometric, and electrochemical [24]. In addition, sensors that utilize the high thermal conductivity of hydrogen, which is about 7.5 times greater than that of air, have also been developed and tested. Such sensors can exhibit a limit of detection down to 500 ppm, while they are also able to operate at temperatures below 0 °C [25]. Last, but not least, sensors that utilize the catalytic reaction of H_2_ with O_2_ to increase the temperature and thus the resistance of the sensing material (known as combustion-based sensors), can exhibit good sensitivity and a linearity for concentrations up to 4% [15].

Electrical sensors that rely on H_2_ absorption (instead of adsorption as in the case of metal oxide semiconductors discussed above) by transition metals have also been proposed and tested [1]. Such sensors are, in principle, more selective compared to those relying on adsorption as not many species can penetrate the lattice of the transition metals. In this respect, Pd, which exhibits high H_2_ solubility, can be employed to attribute high selectivity towards H_2_, thus providing a very promising material for sensing purposes [26,27,28,29,30].

Although materials based on Pd thin films have been successfully synthesized and tested as electrical H_2_ sensors where the conductivity of the sensing material varies proportionally with concentration [31,32], they show certain constraints that can limit their use in real-life applications. At H_2_ concentrations above 2%, for instance, the expansion of the Pd lattice, which is associated to the phase transition of the material (cf. details in Section 2), can degrade the properties of the sensing element, attributing poor irreversibility and stability to the sensor. At lower concentrations (less than 1%), the sensors exhibit long response times due to the slow diffusivity of hydrogen atoms in the crystal lattice, thereby limiting their use for H_2_ sensing.

Sensors that rely on changes of the optical properties of their sensing material upon exposure to H_2_ provide a highly promising alternative to their electrical counterparts. An important advantage of optical sensors is the lack of electrical contacts that could induce sparks under harsh operating conditions, and consequently ignite the sampled gas with catastrophic consequences. This is particularly important when sensing H_2_ in an environment containing O_2_, which is the case for H_2_ sensors designed for safety purposes. Although optical H_2_ sensors reported in the literature have been tested down to a few ppm or less [1,33], in principle they can reach concentrations down to the ppt regime [34].

**Figure 1:**
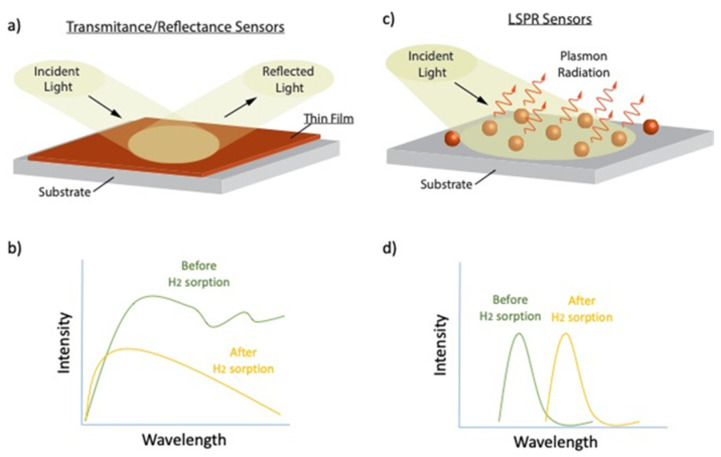
(**a**) Illustration of a thin film structure for reflection (or transmission) H_2_ sensing. (**b**) Graph showing the changes in the optical properties (reflection/transmission) of a thin film-based sensors, upon H_2_ sorption. (**c**) Illustration showing the operating principle of the plasmonic H_2_ sensors composed of isolated nanoparticles. (**d**) Changes in the LSPR radiation spectrum (i.e., peak position and amplitude) for a plasmonic H_2_ sensor comprised of isolated nanoparticles.

Pd-based optical H_2_ sensors (i.e., sensors employing Pd either as the main sensing material or for only catalyzing the dissociation of H_2_ before this is absorbed by the main sensing material) rely on probing either (1) intensity changes of the transmittance/reflectance (cf. Figure 1a,b) [35] or (2) frequency shifts of the localized surface plasmon resonance (LSPR) upon exposure of the sensing element to H_2_ (cf. Figure 1c,d) [36]. Transmission/reflection sensors rely on changes in the optical properties of the sensing material upon exposure to H_2_, which can be easily probed by a simple light detector or a conventional optical spectrophotometer. Although these sensors typically employ the entire visible radiation spectrum (i.e., from 400 to 700 nm), wavelength regimes where the transmission/reflectance change is more intense are used to enhance sensor sensitivity [17,37]. Albeit that such materials can be employed in optical H_2_ sensors, they typically exhibit cracking and hysteresis that deteriorate the overall sensor performance (see discussion further below). In sensors that rely on LSPR peak shifts, metal nanoparticles comprising the sensing element strongly scatter and absorb light due to the collective oscillations of the surface conduction electrons in the resonance frequency. The LSPR peak position depends on several factors, including the size, shape, and the surrounding environment of the nanoparticles employed in the sensing material.

LSPR sensing can occur either in a direct or an indirect manner. In direct LSPR, transition metal nanoparticles deposited on glass substrates play the role of both the active material (used for H_2_ sensing) and the plasmonic signal transducer. The metallic nanoparticles, in this case, form a hydride or a solid solution upon H sorption into the lattice, inducing a subsequent change in their optical properties (shift in the LSPR peak position). Indirect LSPR requires nanomaterials consisting of transition metal (e.g., Pd) nanoparticles, and nanoparticles of a non-H_2_-absorbing element (e.g., Au) that play the role of the plasmonic signal transducer. The absorption of H_2_ by the transition metal nanoparticles (active material) induces a dielectric function change of the non-absorbing nanoparticles and the surrounding environment, yielding enhanced LSPR properties [38,39]. Readers interested in more details on nanoparticle-based LSPR sensors are referred to the recent review paper by Darmadi et al. [40].

Materials that can be used in optical H_2_ sensors typically employ Pd in the form of (1) thin films, (2) materials based on aggregated or isolated nanoparticle (ANP and INP, respectively), or (3) complex nanostructured (CN) architectures. With the term thin films, we refer to continuous flat films, or films that have a negligible surface roughness. ANP-based materials, on the other hand, are films that consist of nanoparticulate building blocks, making them highly porous and providing a roughness that is much higher compared to that of thin films. INP-based materials also employ nanoparticles deposited on a sensor substrate, but in contrast to ANPs, they do not contact one another. Last, CN architectures refer to more elaborate structures, typically consisting of thin films and nanostructured elements such as nanohelices.

Pd-based thin films may exhibit cracking caused by the volume expansion/shrinkage upon hydrogenation/dehydrogenation cycling. Such cracks can affect the optical properties of the films in a non-reversible manner, thereby limiting their application as optical H_2_ sensors. This may be prevented by alloying the Pd and by applying intermediate layers such as Ti on the substrate. Nanoparticle-based materials are much less prone to cracking upon hydrogenation/dehydrogenation, providing a more favorable alternative to thin films. In the case of ANP-based materials, cracks are typically formed during their synthesis (i.e., before exposure to H_2_) [41], providing room for the materials to expand or shrink upon hydrogenation and dehydrogenation, respectively, in a reversible way. For INP-based materials, cracking issues are completely avoided as a result of their non-continuous nature (having freedom levels that expand in all directions), while attributing other highly favorable properties to the resulting sensors in terms of sensitivity and response/recovery times [42,43], as discussed in Section 6.

Hysteresis is another limiting factor in the operation of optical H_2_ sensors that determines their accuracy. The phenomenon is attributed to first-order structural transitions and phase coexistence and is mainly present in thin films due to incoherent phase transformation and plastic deformation. Upon hydrogenation of thin films, their thickness increases due to the addition of H atoms in the Pd lattice, inducing plastic deformation and volume expansion. This plastic deformation enhances the difference between the absorption and desorption isotherms in the hydrogenation/dehydrogenation cycle [44]. It has been shown that employing an alloy (e.g., PdAu), instead of pure Pd, the hysteresis and the response time of the sensors can be improved [45,46]. Combining the advantages of nanostructuring and alloying (e.g., PdAu), a number of studies have reported the fabrication of materials that avoid the hysteresis effect [44,47], and thus improve the overall performance of the sensors.

Another limitation of Pd-based sensors is CO poisoning, which impairs the stability and accuracy of the sensor. At low concentrations, CO adsorbs on specific sites of the Pd lattice that progressively covers the entire material [48]. Eventually, a C layer is formed on the surface of the Pd material, forming a barrier for H_2_ to reach the Pd surface. PdAuCu and polymeric coating can be applied to prevent CO poisoning as discussed in more detail in Section 4.

This paper provides an overview of Pd-based thin films, nanoparticle-based materials, and complex nanostructured architectures that can be employed in optical H_2_ sensors. Specifically, we describe the operating principles of sensors employing materials that exhibit changes in transmittance/reflectance, which is the first category of optical sensors, or shifts in LSPR peak position, which is the second category of optical sensors reviewed here. In addition, we discuss the main limitations of each material type and approaches towards overcoming them.

The rest of the paper is structured as follows. Section 2 provides a brief theoretical background on Pd-H thermodynamics and the resulting optical changes of the system. Section 3 focuses on H_2_ sensors that employ thin films, including bi-layered structures, whereas Section 4 gives relevant information on ANP- and INP-based materials. Section 5 provides a brief overview of complex nanostructured materials for optical H_2_ sensors, whereas Section 6 summarizes and highlights the pros and cons of the different material categories.

## 2. Theoretical Background

**Figure 2:**
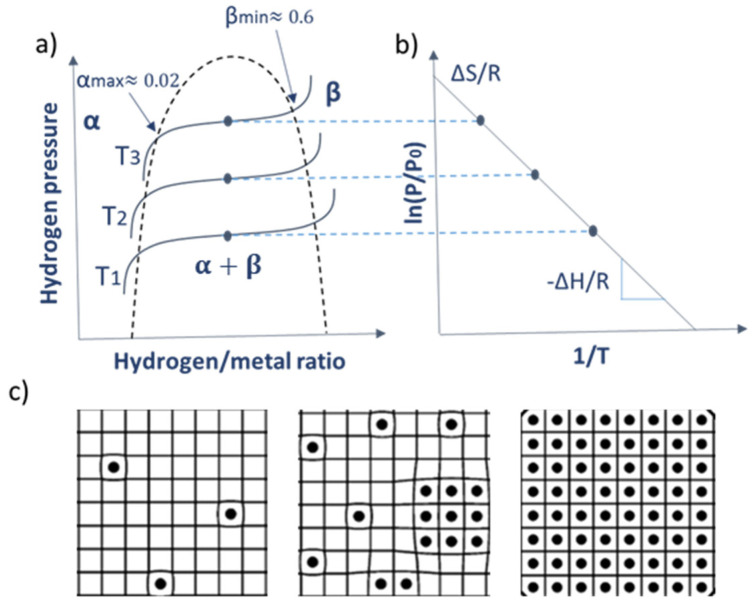
(**a**) Pressure—Composition-Temperature (PCT; where T_1_ < T_2_ < T_3_) isotherms of the ideal Pd-H system including the regions corresponding to the three phases (α, β, and α + β) of the system, and the temperature-dependent plateau that disappears at high temperatures. (**b**) Van’t Hoff diagram corresponding to the isotherms of Figure 1a. (**c**) Illustrations showing the fraction of H atoms (black dots) in the Pd interstitial sites for the different phases: α phase (left), α + β phase (middle), and β phase (right).

Figure 2a shows a schematic representation of an ideal pressure-composition-temperature (PCT) phase diagram of the Pd-H system. At low H_2_ pressures, the system forms a solid solution of H in the metal host corresponding to the α phase. At high H_2_ pressures the Pd metal is transformed to PdH_x_, forming the β phase, whereas at intermediate H_2_ pressures, the α and β phase coexist. The latter mixed phase exhibits an equilibrium plateau corresponding to the hydride formation equilibrium pressure in the isotherm diagram, which disappears above the critical temperature (cf. Figure 2a). The plateau describes the pressure of the first-order phase transition between two solid solutions; one with a low and the other with a high hydrogen content, forming a completely different phase (i.e., the α + β phase).

The temperature dependence of the equilibrium pressure in the mixed-phase is given by Van ’t Hoff’s equation, which can be expressed as

(1) $$\ln\frac{p}{p_{0}}=\frac{\Delta H}{\mathrm{RT}}+\frac{\Delta S}{R}$$

where p is the equilibrium H_2_ plateau pressure; p_0_ is the standard pressure; ΔH and ΔS are, respectively, changes in enthalpy and entropy of hydrogen when forming a solid solution phase; R is the gas constant; and T is the temperature. The variability of both the entropy and enthalpy with H_2_ concentration is critical for the overall performance of optical H_2_ sensors, including their desorption activation energy barrier that can be determined by ΔH as a first approximation [49].

The lattice constant in the α phase increases by up to 0.1% compared to that of pure Pd metal (from 3.890 to 3.895 Å), whereas a lattice constant change of up to 3.5% (from 3.895 to 4.029 Å) occurs in the mixed phase. An additional increase in the lattice constant (by up to 0.1%) takes place in the β phase. We should note here that upon the monoatomic hydrogen absorption by Pd, its lattice constant changes in a linear manner.

The relation between the thermodynamics of Pd-H thin films and their optical properties is given by Lambert´s law:

(2) $$T\left(x,\lambda ,z\right)=\frac{I\left(z\right)}{I_{0}}e^{-\mu \left(x,\lambda \right)z}$$

where T is the transmittance of the hydride sensing material; I and I_0_ are, respectively, the intensities (or radiant flux) of the transmitted and incident light through/on the sensing material; μ(x,λ) represents a wavelength-dependent attenuation coefficient; x and λ are the attenuation length and light wavelength, respectively; and *z* is the thickness of the layer. The attenuation coefficient is given by

(3) $$\mu (x,\lambda )=(1-f)\mu_{1}(x_{1},\lambda )+f\mu_{2}(x_{2},\lambda ),$$

where f = (x − x_1_)/(x_2_ − x_1_) = Δx/(x_2_ − x_1_), x_1_ and x_2_ are the fractions of H in the solid solution of the two-phase system, whereas μ_1_ and μ_2_ are the attenuation coefficients corresponding to the α and the β phase, respectively. Equation (3) can be applied when the hydrogen sorption attenuation effect does not depend on the hydrogen concentration in the lattice of Pd. Although Equation (3) extends for the whole range of x values in PdH_x_, strictly speaking it is not valid for the hydrogen absorption only in one phase. Substituting Equation (3) in Equation (2) yields [37]

(4) $$\ln\left(\frac{T\left(x,\lambda ,z\right)}{T_{0}}\right)=-c\left(\lambda \right)z\Delta x.$$

where T_0_ = $e^{-\mu_{1}\left(x_{1},\lambda \right)z}$ is the transmission of the α phase, and c(λ) = $-\frac{1}{x_{2}-x_{1}}$($\mu_{2}\left(x_{2},\lambda \right)-\mu_{1}\left(x_{1},\lambda \right)$) is a wavelength-dependent factor that can be determined by the attenuation coefficients corresponding to the α (μ_2_(x_2_,λ)) and the β (μ_1_(x_1_,λ)) phase. Equation (4) shows that there is a linear relationship between ln(T(z)/T_0_) and H concentration, through the dependence of the wavelength constant *c* on the attenuation coefficients of the sensing material. Although this has been verified for Pd, it has not been confirmed for other material systems [37].

## 3. Pd-Based Thin Films for Optical H_2_ Sensors

The most investigated family of materials for optical H_2_ sensing is that of Pd thin films (i.e., films having thicknesses from few tens to few hundreds of nanometers) deposited on flat transparent substrates. In such films, Pd has a double role: (1) it acts as a catalyst to dissociate molecular hydrogen and (2) it changes its reflection/transparency upon hydrogen absorption, which is the property probed to determine the concentration of H_2_ in the sample. Pd thin films can be produced by conventional methods including Physical Vapour Deposition (PVD) or sol–gel techniques [50,51,52,53,54,55].

Pure Pd thin films, deposited on SiO_2_ substrates, have been proposed as optical material for sensing H_2_ since the late 1980s [56]. The transition from the α to the β phase that can induce a lattice volume increase can potentially cause material deformation and cracking. In principle, this is seen as a disadvantage because it can lead to a non-repeatable behavior of the sensor. Nevertheless, deformations and cracks of Pd thin films can be used to optimize the performance of the sensing elements when exposed to a H_2_-containing gas as they can increase the surface to volume ratio of the material [50,51,57]. For example, reduced Pd thin films have been shown to exhibit pronounced changes in their optical properties compared to their oxidized counterparts, because cracks increase the available sites for absorption upon reduction [50,51].

In an attempt to improve the performance of Pd thin film sensors in terms of sensitivity and limit of detection, a number of studies have tried to cap an elastomer with a Pd thin film. A Pd-capped elastomer (PCE) sensor exploits the deformation of the sensing element to radically change the absolute reflectance, showing up to ~60% increase in the reflectance compared to samples without an elastomer, over the entire visible spectrum when exposed to air containing 4% H_2_ [58]. This material deformation changes the specular (mirror-like reflection) surface of the Pd thin film to a diffusing (where incident rays are scattered in many angles) surface, thereby enhancing the light scattering efficiency of the material upon absorption of H_2_. Sensors employing PCE materials have been reported to have a response time of 14 s, when exposed to 4% H_2_ in air, and a recovery time of 10 s [58].

An important ability of Pd thin films is that they can dissociate molecular into monoatomic hydrogen. Using this capability, Pd thin films have been employed as a top layer of bi-layer structures for optical H_2_ sensors [59,60,61]. In such bi-layer structures, monoatomic hydrogen produced by the dissociation of H_2_ at the surface of the Pd thin film is subsequently absorbed by a second layer typically consisting of oxides or transition metals that play the role of the sensing elements. Such material architectures can support improved sensing performance in terms of intense optical changes and detection limits compared to pure Pd systems [59].

**Figure 3:**
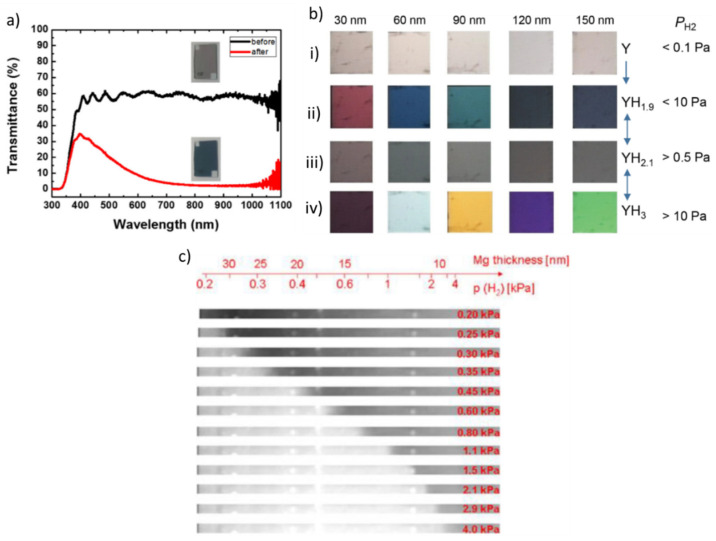
(**a**) Transmittance spectra of a 760 nm thick WO_3_ layer, capped with a Pd layer of 4 nm, before (black curve) and after (red curve) 10 min exposure to 1% H_2_ in air containing oxygen, water vapors, and nitrogen. Reproduced from [60], with permission from Elsevier, 2017. (**b**) Colors of Pd-capped Y thin films that have been exposed to different concentrations of H_2_ at room temperature. Adapted from [37], with permission from JPS, 2020 (**c**) Colors and resulting thickness of Pd-capped Mg films exposed to H_2_ pressures ranging from 0.2 to 4 × 10^3^ Pa. Mg becomes highly transparent when converted to MgH_2_. Reproduced from [64], with permission from Elsevier, 2010.

Pd-capped WO_3_ provides an excellent bi-layer thin-film material architecture for transmission/reflection H_2_ sensing [60,61]. In a H_2_-containing environment, the H_2_ adsorbed on the surface of the Pd thin film dissociates and diffuses to the WO_3_ layer which is converted to tungsten bronze (HWO_3_), exhibiting a noticeable change in its optical properties as it becomes opaque (dark blue) [60,62]. In those systems (also referred to as gasochromic sensors), the thickness of the Pd layer should be small enough (~3–4 nm) to ensure high optical transparency. Figure 3a shows that the transmittance of a 760 nm thick Pd-capped WO_3_ film before and after 10 min exposure to 1% H_2_, as reported by Lee et al. [60], can exhibit a large change in the visible and near-infrared regime.

Other Pd-capped thin films that can provide promising optical H_2_ sensors employ Yttrium (Y) and Magnesium (Mg) as capped materials [63,64,65]. These elements undergo a metal to semiconductor transition upon hydrogenation, inducing color changes by interference effects. Figure 3b shows the color of Pd-capped Y thin films (having thicknesses that range from 30 to 150 nm) at different levels of hydrogenation (Y→YH_1.9_→YH_2.1_→YH_3_). We should note here that Y reacts with oxygen if not capped, becoming transparent upon oxidation. As a result, by capping it with Pd in principle offers good selectivity towards H_2_ when this is present in an oxygen-containing environment. Color changes in the YH_1.9_→YH_2.1_→YH_3_ states correspond to H_2_ threshold concentrations ranging from 5 to 1000 ppm, whereas the time required for these transitions can vary from 10 to 100 s depending on the concentration of H_2_ in the system, which can be considered too slow for certain applications. In addition, those systems are strongly hysteretic, posing another limitation for their use.

Similar to Y, Mg films also exhibit limitations for optical H_2_ sensing that can be overcome by capping them with Pd. Mg exhibits optical transition within a narrow pressure range around the pressure plateau (as illustrated in Figure 2a) that limits the sensitivity of the resulting sensors, but this can be overcome by doping it with other elements, such as Ni, Ni-Zr, and Ti [66]. Moreover, Mg also shows strong hysteresis upon hydrogenation/dehydrogenation cycling [64], mainly associated to the complex phase transformation, which is also responsible for the slow response and relatively high inaccuracy of the resulting sensors. Despite that, Pd-capped Mg thin films can be a choice for a single-use eye-readable H_2_ sensor (cf. Figure 3c).

Pd-capped transition metals, such as Hf and Ta, have also been proposed as H_2_ sensing materials. Hf exhibits steeper optical changes over a wider H_2_ pressure range, whereas Ta shows less abrupt changes at low H_2_ pressure (<10^−1^ Pa), compared to those of pure Pd or PdAu thin films. In the case of Hf, the material does not exhibit hysteresis upon hydrogenation/dehydrogenation cycling, due to the occurrence of a coherent transition from HfH_1.4_ to HfH_2_ (referred to as the *δ* to *ε* transition) [67,68]. This transition appears at the range where the optical transmission spans six orders of magnitude in pressure, attributing a very high spanning range to the resulting sensor. In general, Pd-capped Hf thin films exhibit a response time that ranges from ~5 s to 10 min as the H_2_ pressure decreases from 5 to 10^−2^ Pa at 120 °C. Note here that the lowest H_2_ pressure tested was due to limitations of the testing setup and not of the sensing material.

**Figure 4:**
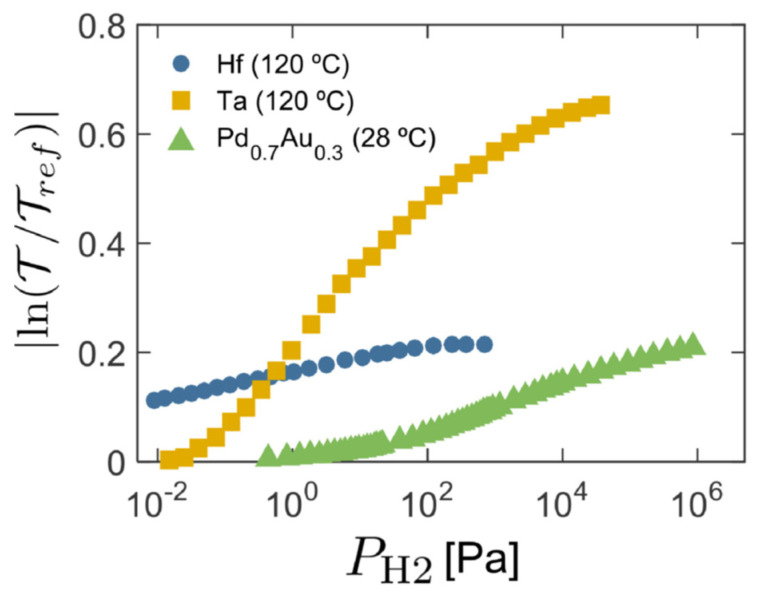
Optical contrast over a wide H_2_ concentration for a Pd-capped Hf and Ta thin films, in comparison to the contrast exhibited by a Pd_70_Au_30_ film of similar thickness. In the cases of the Pd-capped Ta and the Pd_70_Au_30_ thin films, a 4-nm Ti layer is used as an adhesion layer. Reproduced from [67], with permission from Elsevier, 2019.

Ta shows a higher H_2_ solubility compared to Hf, associated with the absence of any structural changes upon hydrogenation. Pd-capped Ta thin films have demonstrated a repeatable hysteresis-free optical response in the range of 10^−2^ to 10^4^ Pa (cf. Figure 4), with the intensity of the optical change being wavelength-dependent. Such films have also been reported to have response and recovery times of 7 and 20 s, respectively, when exposed to H_2_ pressures ranging from 10 to 300 Pa. In general, Ta-based thin films exhibit short/sub-second response times at close to room temperatures compared to their Hf-based counterparts, large H_2_ sensing range [59], making them highly attractive for many applications.

## 4. Nanoparticle-Based Materials for Optical H_2_ Sensors

There are two types of nanoparticle-based materials that can be employed for optical H_2_ sensing: namely, those made of aggregated nanoparticles (ANPs), and those that use isolated nanoparticles (INPs). In the first type of materials, nanoparticle building blocks are used to form porous thin films, also referred to as nanoparticulate thin films, that exhibit changes in their transparency/reflectance upon exposure to H_2_. In the second type of materials, nanoparticles deposited on glass substrates without touching each other are illuminated to probe shifts in the LSPR that are proportional to the concentration of H_2_ in the gas they are exposed to. The paragraphs that follow provide an overview of these two types of nanoparticle-based materials.

### 4.1. Materials Based on Aggregated Nanoparticles

Materials that employ ANPs exhibit some unique advantages compared to conventional (or continuous) thin films discussed in Section 3. First, they have a high surface to volume ratio, which in principle can enhance the performance of the sensor by improving its sensitivity and response/recovery time if the surface is a limiting factor. By reducing the size of the nanoparticle building blocks one can increase the available sensing surface, which in turn can accelerate the monoatomic hydrogen flux within the Pd lattice. Moreover, the reduced volume of the smaller nanoparticles can further decrease the diffusion path of hydrogen atoms to the center of the nanoparticles, allowing them to reach faster a new equilibrium state [69]. The smaller the nanoparticle building blocks the shorter the response times of the resulting sensors for a wide concentration range of hydrogen [33].

Another advantage of ANP-based materials is that they exhibit a hysteresis-free behavior by alloying, reducing the size of the nanoparticle building blocks, as well as by local heating of the sensing elements [40]. For example, films of PdAu ANPs exhibit a hysteresis-free behavior when their thickness is below a certain threshold (~200 nm). Although a combination of effects can explain the reduced response time and hysteresis, further work is required to understand the mechanisms leading to this observation and consequently for improving the design of the sensing materials. A thorough discussion on the ways of reducing hysteresis and improving response times are provided by Darmadi et al. [40].

**Figure 5:**
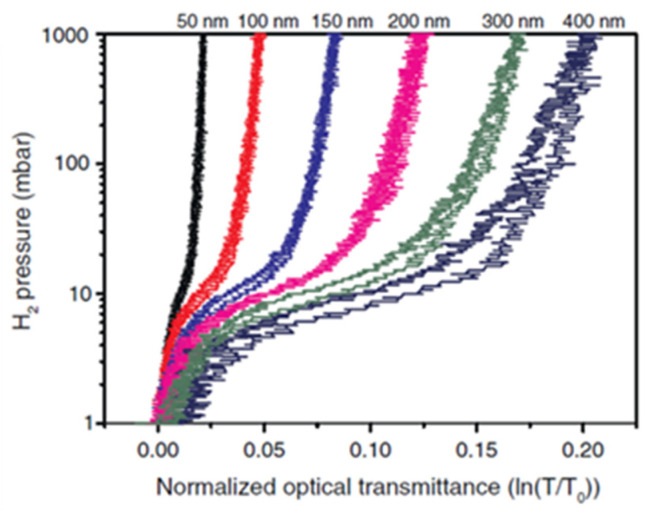
Pressure transmission isotherms for nanoparticulate Pd_88_Au_12_ thin films with thicknesses ranging from 50 to 400 nm. Adapted from [41], with permission from Elsevier, 2015.

Aerosol-based techniques are among the few that have been used to produce ANP-based optical H_2_ sensors. More specifically, spark ablation has been employed to synthesize well-defined PdAu nanoparticles, which were subsequently immobilized on flat surfaces by inertial deposition to form nanoparticulate thin films [41,70]. Using Pd_88_Au_12_ ANPs, Isaac et al. [41] synthesized thin films that exhibit a hysteresis-free response when their thickness is smaller than ~200 nm (cf. Figure 5). This is in contrast to continuous thinner films (40 nm) of very similar composition (Pd_85_Au_15_), which exhibit hysteresis [44]. Despite that other aerosol-based methods (e.g., flame synthesis [20,71,72,73,74,75,76]) can be used to produce Pd-based nanoparticulate sensors, so far only spark ablation has been used to produce ANP-based materials for optical H_2_ sensors.

Apart from controlling the thickness of the ANP-materials, aerosol-based methods offer the advantage of controlling the size of the nanoparticle building blocks. This has been demonstrated in a recent work where monodisperse Pd-C core–shell particles were produced by aerosol-based methods (i.e., spark ablation) to build materials for electrical H_2_ sensors [77]. Given the first promising results showing that ANP-based materials can offer certain advantages compared to their thin-film counterparts, further work is required to explore how their intrinsic properties (e.g., size of nanoparticle building blocks, material porosity, etc.) can affect the performance of the resulting sensors.

Nanoparticulate-like thin films can also be synthesized by oblique angle deposition under vacuum (a PVD technique) if the thickness of the layer is small (i.e., a few nm). This is due to the role that surface tension plays in thin film synthesis, resulting in the formation of nanoparticles that become agglomerates to form ANP-based structures on the substrate. William et al. [78] showed that this method can be employed to fabricate ultrasensitive optical sensors that can measure H_2_ at pressures from 10^−1^ to 10^3^ Pa, whereas the response times of the resulting sensors range from 0.6 to 50 s as the concentration decreases from 4% to 50 ppm of H_2_ in Ar [78].

### 4.2. Materials Based on Isolated Nanoparticles

In contrast to ANP-based, INP-based materials employ alloy or heterodimer nanoparticles that are not in contact with each other, relying on probing shifts of the LSPR peak when they are exposed to a H_2_-containing atmosphere. INPs can be placed in ordered array structures by classical lithography (i.e., using prefabricated masks) [79], or by shrinking-hole colloidal lithography [80]. Alternatively, INPs synthesized by aqueous solutions/colloids can randomly be deposited on substrates to fabricate plasmonic sensors [81,82].

**Figure 6:**
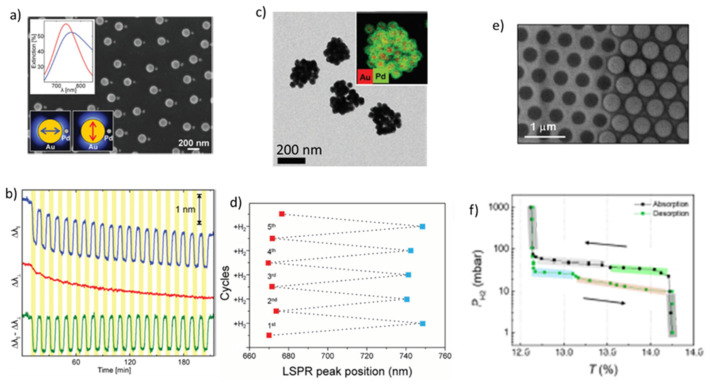
(**a**) SEM image and illustration of a PdAu heterodimer plasmonic H_2_ sensor. The inert plasmonic Au particles have a diameter of 140 nm, while the Pd nanoparticles have a diameter of 40 nm. The top inset shows extinction spectra for the two polarizations. The red line corresponds to the perpendicular, while the blue line corresponds to the parallel polarization. The bottom inset shows a schematic illustration of the two polarizations. (**b**) PdAu heterodimer sensor response upon exposure to 4% H_2_ in air. The blue and red curves correspond to LSPR peak change during the excitation in the parallel and perpendicular polarization, respectively, while the green curve represents the difference between them. White vertical areas correspond to periods when the material is exposed to pure Ar, while yellow areas to Ar containing 4% H_2_ at 30 °C. Reproduced from [80], with permission from The Royal Society of Chemistry, 2015. (**c**) STEM images of Au-Pd core-shell nanoparticles with an average shell thickness of 9 nm. Inset shows the EDS mapping from STEM. (**d**) LSPR changes upon 5 hydrogenation/dehydrogenation cycles of a material containing Au-Pd core–shell nanoparticles. Adapted from [83], with permission from Wiley, 2018. (**e**) SEM image of the BPNL structure. (**f**) Example of H_2_ absorption–desorption isotherms of BPNL structures. Reproduced from [85], with permission from Elsevier, 2020.

In optical LSPR H_2_ sensors that employ heterodimer nanostructures of Pd and Au particles, the former element acts as an active analyte (i.e., the particle used for the H sorption) and the latter as a nanoantenna, exhibiting a dominant and strong LSPR [80]. Figure 6a shows an SEM image of INP-based materials and its optical characteristics (inset) for optical H_2_ sensing. Drifts in the LSRP can be produced either by non-specific events (e.g., temperature change) or by the sensor material itself. To provide a reliable measurement, one has to ensure that the smaller Pd particles are placed next to larger Au nanoparticles [80]. Figure 6b shows results from cycling experiments with a plasmonic H_2_ sensor employing PdAu heterodimer nanostructures when alternating between 0 and 4% H_2_ in Ar at 30 °C. Unwanted contributions induced by surface contamination and/or potential temperature changes can be removed by subtracting the curve corresponding to the incident light polarization that is perpendicular to the axis formed by the two nanoparticles (red curve) from that corresponding to the incident light polarization that is parallel to the axis formed by the two nanoparticles (blue curve), and thus H sorption remaining the only process altering the optical properties of the material.

Another family of INP-based materials is that employing Au-Pd core–shell nanoparticles (Figure 6c) [83]. Interestingly, the thicker the Pd shell, the higher the amount of absorbed monoatomic hydrogen, as there is more Pd that can be transformed to the PdH_x_. Au-Pd core–shell nanoparticles with an average Pd shell thickness of 9 nm can show a shift in LSPR wavelength peak (Δλ_peak_) of 78 nm. Figure 6d shows changes in LSPR over 5 cycles of H_2_ exposure/release. The experimental results also show that the Au-Pd core-shell nanoparticles (produced by wet chemistry techniques) exhibit a reversible behavior, similarly to the case of PdAu alloy nanoparticles that are discussed below [83]. Nanobipyrapid Au-Pd core–shell structures with different Pd shell thicknesses (produced again by wet chemistry techniques) have also been shown to exhibit a large redshift of 140 nm at 2% H_2_ [84].

Another promising INP-based material architecture is that of bilayer plasmonic nanolattices (BPNL), consisting of Pd nanoparticles and nanoholes placed adjacent to one another [85]. In this case, the nanoparticles support the LSPR, whereas the nanoholes provide surface plasmon polaritons, i.e., evanescent electromagnetic waves traveling across a metal-dielectric/air interface [86]. In such sensors, which have been fabricated by electron beam deposition and reactive ion etching [85], the optical properties of the sensing material depend on hole size, nanoparticle diameter, as well as the distance between the nanoparticles and nanoholes.

Figure 6e,f shows, respectively, a top view of Pd nanoparticles/nanoholes and changes in the transparency of a BPNL structure during a hydrogenation/dehydrogenation cycle. In general, the response time of the BPNL structure decreases with decreasing H_2_ pressure. The response time can be below 100 s at the peak resonance, corresponding to a wavelength of 460 nm, when the H_2_ pressure decreases from 4 × 10^3^ to 10^3^ Pa. The sensitivity of the BPNL sensor can be up to 2 and 4 times higher compared to systems consisting only of nanoparticles or nanoholes, respectively, as reported by Luong et al. [85].

To improve the performance of LSPR sensors, Strohfeldt et al. [87] investigated the sensing abilities of multilayer PdAu nanodiscs. The LSPR peak position of these nanodiscs is determined by the bottom layer. When Au is at the bottom, the sensors exhibit a higher sensitivity. We should also note that the Pd_75_Au_25_ alloyed nanodiscs (deposited on flat surfaces or optical fibers) exhibit a hysteresis-free response [45]. These nanodiscs have been tested in the concentration range from 10^2^ to 10^5^ Pa, with a response and recovery time being strongly dependent on the Au content. For example, the optimized composition of Pd_75_Au_25_ can support a response time of about 1 s.

**Figure 7:**
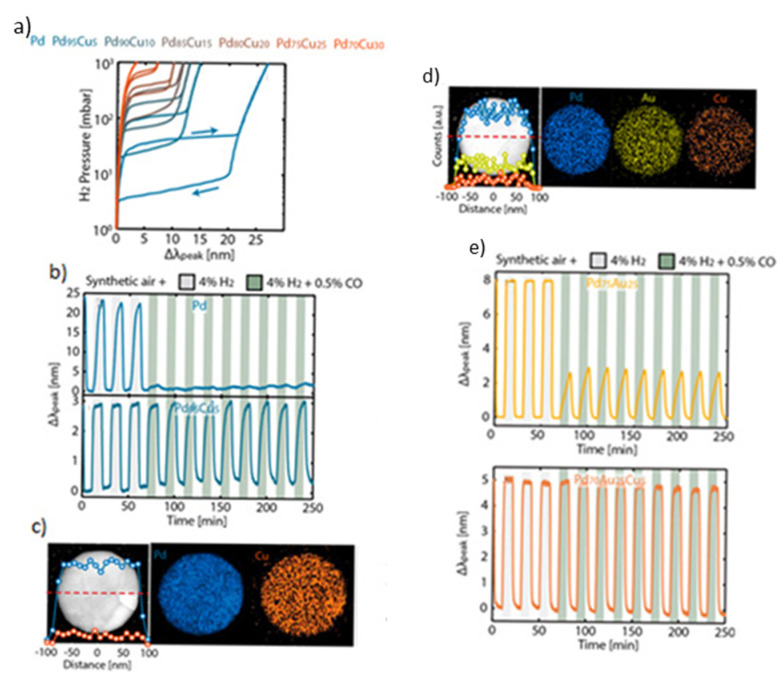
(**a**) Optical response of INP-base material with PdCu alloy nanoparticles when exposed at pressures between 1 and 10^3^ mbar (10^2^ to 10^5^ Pa). The right and left arrows indicate the absorption and desorption part of the cycle. (**b**) Hydrogenation/dehydrogenation cycling in Pd and Pd_95_Cu_5_. In both cases, the first three gray-shaded areas correspond to 10-min exposure periods of the sensing material to 4% H_2_. In the remaining nine green-shaded periods, the sensing material was exposed to a gas consisting of 4% H_2_ + 0.5% CO at ambient pressure. (**c**) STM image and its corresponding EDS line-scan (red dashed line; left), and elemental mapping (right) of a Pd_85_Cu_15_ nanodisc. The blue data points (left) and mapping (right) correspond to the fraction of Pd of the nanodisc, while the orange points (left) and mapping (right) represent the Cu fraction of the nanodisc. (**d**) STM image of a Pd_70_Au_25_Cu_5_ nanodisc (left) and the corresponding EDS elemental mapping (right). The blue data points (left) and mapping (right) correspond to the fraction of Pd, the yellow points (left) and mapping (right) to the Au fraction, and the orange points (left) and mapping (right) to the Cu fraction. (**e**) Hydrogenation/dehydrogenation cycling in Pd_75_Au_25_ and Pd_70_Au_25_Cu_5_. In both cases, the first three gray-shaded areas represent the periods when the materials were exposed to synthetic air containing 4% H_2_. The remaining 9 areas correspond to periods when the material was exposed to synthetic air containing 4% H_2_ and 0.5% CO. Adapted from [79], with permission from ACS Publications, 2019.

Several studies have investigated the possibility of using alloy nanoparticles, instead of single-component nanoparticles, composed of two elements in LSPR sensors. Figure 7a illustrates the dependence of Δλ_peak_ shift on H_2_ pressure upon hydrogenation/dehydrogenation for a material consisting of PdCu alloy nanoparticles, whereas Figure 7c shows their elemental mapping. Evidently, the higher the Cu concentration in the alloy nanoparticles, the lower the sensitivity of H_2_ pressure on the Δλ_peak_ shift, the smaller the pressure equilibrium plateau, and the more pronounced the hysteresis effect (Figure 7a). Despite that, the materials consisting of Pd_70_Cu_30_ nanoparticles being an optimum solution that exhibits a hysteresis-free behavior. An important advantage of PdCu alloy nanoparticles is the enhanced resistance against CO poisoning. More specifically, Pd_95_Cu_5_ nanoparticles can present remarkable resistance to CO poisoning (Figure 7b, bottom graph), compared to pure Pd that exhibits fast degradation upon exposure to a CO-containing gas (Figure 7b, top graph) [79].

PdAuCu alloy nanoparticles (cf. Figure 7e) attribute to the sensors several advantages, including a hysteresis-free behavior, a low limit of detection, and great resistance against CO poisoning (Figure 7d) [88,89]. In general, Pd_70_Au_25_Cu_5_ showed the closest performance to the optimized nanoparticle system of Pd_75_Au_25_ in terms of sensing and hysteresis-free response [45]. In addition, the Pd_70_Au_25_Cu_5_-particle-based sensor is more sensitive than those employing Pd_70_Cu_30_ particles, as it exhibits a stronger Δλ_peak_ change upon exposure to 4% H_2_. What is more, a response time of 0.4 s is obtained for these sensors when exposed to 4000 Pa H_2_ in vacuum conditions, which is two orders of magnitude lower compared to that of sensors employed pure Pd particles (i.e., 42.2 s).

**Figure 8:**
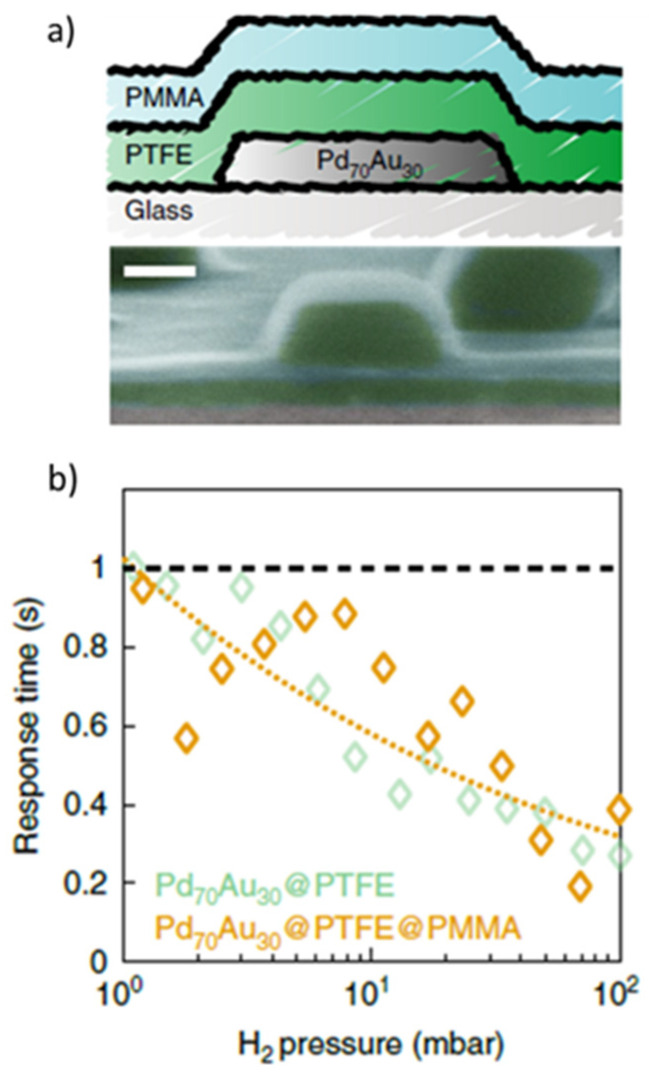
(**a**) Cross section (top) and SEM image (bottom; scale bar of 100 nm) of a Pd_70_Au_30_@PTFE@PMMA nanodisc-based tandem sensor. (**b**) Response time as a function of H_2_ pressure when the material was coated only with PTFE or with PTFE and PMMA. The dashed orange line represents a power-law fit through the data corresponding to the Pd70Au30@PTFE@PMMA. Reproduced from [33], with permission from Nature Publishing Group, 2019.

Another approach for reducing CO poisoning, while maintaining low response times, is to employ polymeric coatings on the INP-based materials. Such an example comes from Nugroho et al. [33] who fabricated nanodiscs coated with layers of PTFE and PTMA. Figure 8a shows a cross section and an SEM image of the proposed Pd_70_Au_30_ tandem PTFE (30 nm)@PMMA(35 nm) structure. The improved performance of this material compared to those without any polymeric coating, is mainly related to the reduction of the activation energy barriers in Pd, through H diffusion via a polymeric-metal surface bond formation. The response times of these materials over a wide range of H_2_ partial pressures (typically less than 1s when exposed to 1 mbar H_2_ at ambient temperature) are very similar to those when the PMMA capping layer is not used (Figure 8b).

## 5. Complex Nanostructured (CN) Materials for Optical H_2_ Sensors

**Figure 9:**
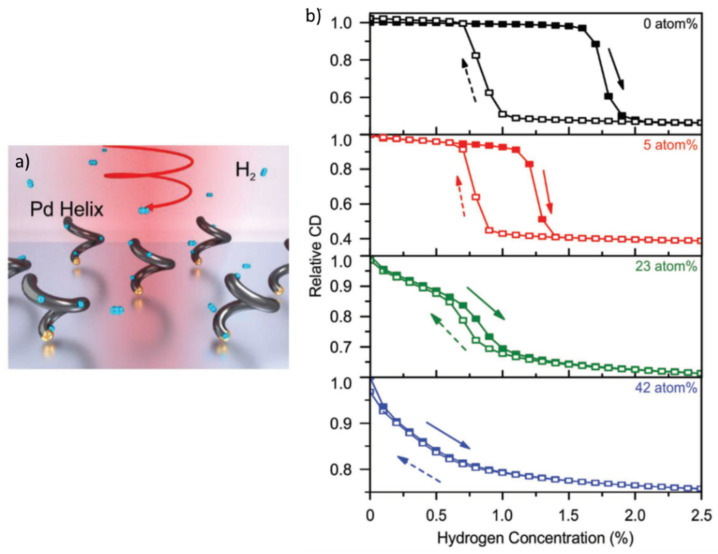
(**a**) Schematic illustration of plasmonic nanohelices for H_2_ sensing. (**b**) Circular dichroism signal of PdAu nanohelices, having different Au fraction, as a function of H_2_ concentration. Reproduced from [90], with permission from Wiley, 2018.

More complex material architectures compared to thin films or ANP/INP-based materials typically require elaborate fabrication processes. Nanohelices, for example, can be employed for circular dichroism spectroscopy for high-sensitivity H_2_ sensing [90]. Such structures can be synthesized by a number of techniques including nano-glancing angle deposition (Figure 9a). Similarly to the approaches followed for conventional thin films or ANP- and INP-based materials, the hysteresis effect exhibited by the PdAu nanohelice can be decreased by increasing the fraction of Au. An optimized performance has been reported for Pd_77_Au_23_ nanohelices (Figure 9b), attributing toe the resulting sensors an almost hysteresis-free behavior and a response time slightly less than 20 s when exposed to 2.5% H_2_ in N_2_. The response times of nanohelice-based sensors can reach values even below 1 s, by optimizing the crystallinity and the alloy mixture [90].

**Figure 10:**
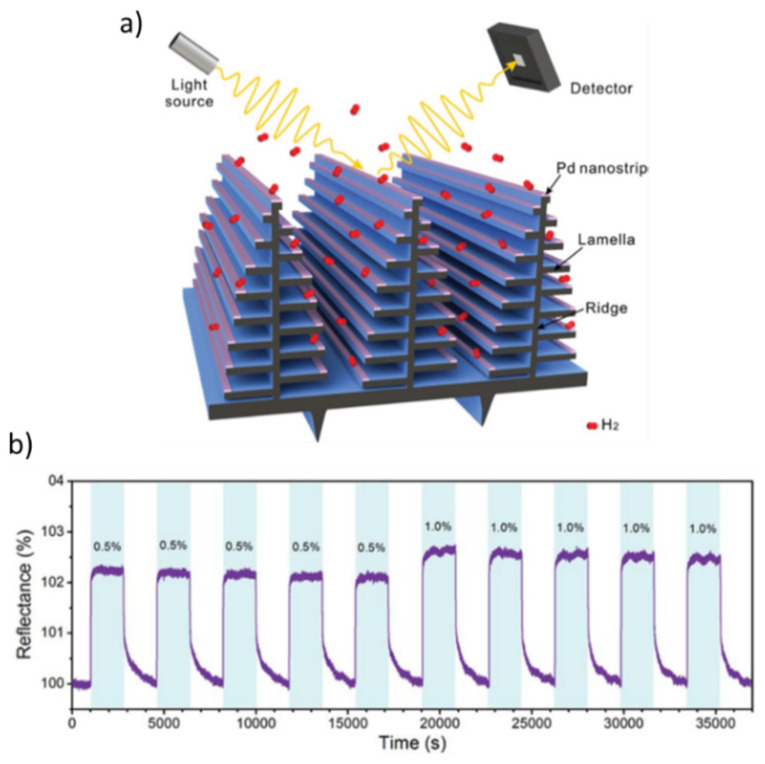
(**a**) Schematic illustration of an optical H_2_ sensor based on 3D photonic structures consisting of Morpho butterfly wings and Pd nanostrips. (**b**) Five cycles during which the Morpho butterfly wing structure is exposed to either 0.5 or 1.0% H_2_ and a H_2_-free gas. Reproduced from [91], with permission from The Royal Society of Chemistry, 2018.

Another material architecture for optical H_2_ sensing is that based on 3D-photonic structures consisting of Morpho butterfly wing scales, decorated with Pd nanostrips (cf. Figure 10a) [91]. Such nanostructured materials can couple the plasmonic mode of the Pd nanostrips and the optical resonance of the photonic crystal Morpho butterfly, thereby attributing a high sensitivity (0.5% at 0.001% of H_2_) to the resulting sensor. These nanostructures can exhibit an intense reflectance peak in the blue regime of the electromagnetic spectrum, which can shift upon H sorption by the Pd nanostrips. Figure 10b shows five cycles during which a Morpho butterfly material was interchangeably exposed to either 0.5 or 1.0% H_2_ in N_2_. These materials can exhibit a limit of detection of 10 ppm and relatively high response times (e.g., ~15 s at 1.0% H_2_) [91].

**Figure 11:**
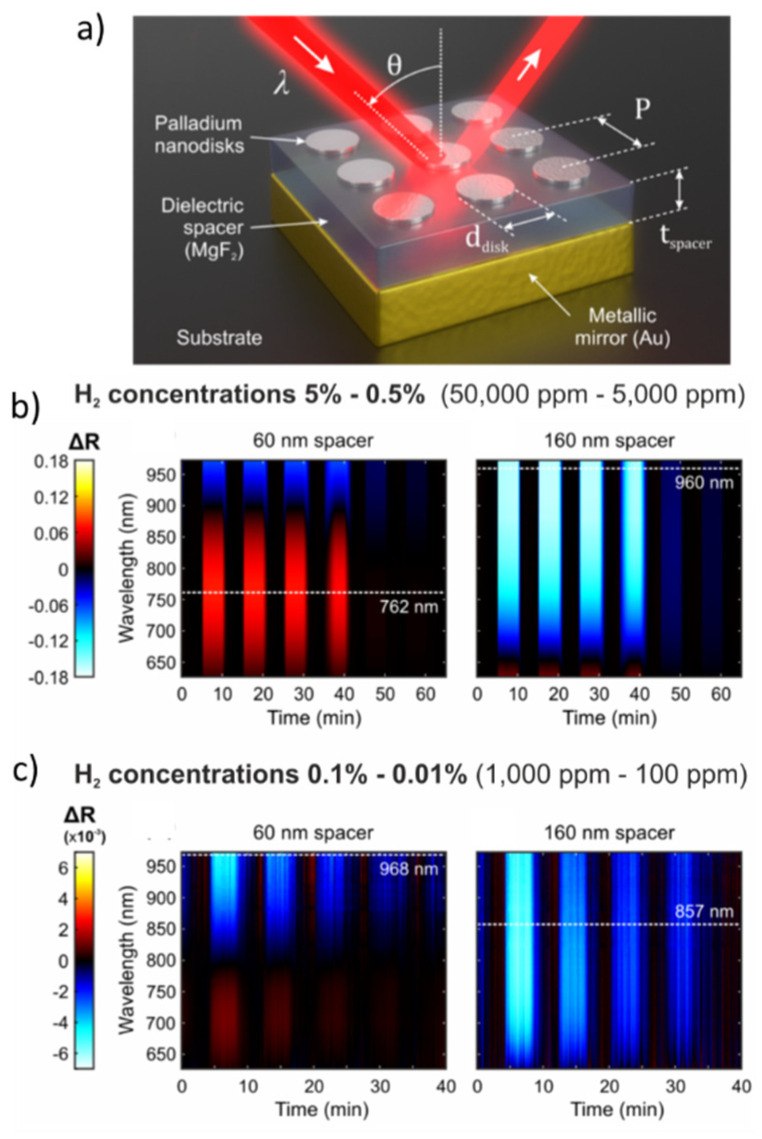
(**a**) Schematic illustration of the plasmonic perfect—absorber—based H_2_ sensor. (**b**) Reflectance of perfect-absorber materials having spacers with a thickness of 60 nm (left graph) and 160 nm (right graph). Each vertical colored line (blue and red in the left graph, and light blue in the right graph) corresponds to a period when the materials are exposed to a fixed H_2_ concentration. Dashed white lines show the maximum reflectance difference. (**c**) As in panel (b) but for reflectance measurements upon cycling H_2_ concentrations ranging from 0.1 to 0.01%. Reproduced from [92], with permission from ACS Publications, 2020.

Another family of CN materials are those that combine layers of thin films and arrays of isolated nanodiscs. These materials, referred to as plasmonic perfect-absorber-based H_2_ optical sensors [92,93,94,95], exploit changes in the reflectance during hydrogenation/dehydrogenation [92]. An example structure of such a material comprises of an array of Pd nanodiscs that is placed on top of a dielectric spacer (MgF_2_) while the bottom layer is a metallic film (e.g., Au), which plays the role of a mirror (Figure 11a). The perfect-absorber exhibits near unity absorption in the absence of H_2_ in the overlaying gas, which is strongly related to the size of the disks, the distance between them, and the thickness of the dielectric spacer (MgF_2_).

Figure 11b shows the reflectance difference (ΔR) as a function of time and wavelength for the two perfect-absorber materials having dielectric spacer thicknesses of 60 (left spectrum) and 160 nm (right spectrum). These spectra correspond to H_2_ concentrations ranging from 0.5 to 5% in N_2_. The dotted lines in Figure 11b represent the wavelength at which the materials exhibit the highest sensitivity. Figure 11c shows similar results when the materials are exposed to lower H_2_ concentrations (from 0.1% to 0.01%). Despite that the reflectance differences are limited in this concentration range, the materials can still produce a detectable signal that can be used to determine the concentration of H_2_ in the overlaying gas. As reported by Sterl et al. [92], the response times of the perfect absorbers is in the order of a few seconds when the materials are exposed to H_2_ concentrations of a few percent, but increases to a few minutes when this drops to the ppm range.

## 6. Discussion

All materials for optical H_2_ sensors considered here are based on the ability of Pd to dissociate molecular hydrogen into monoatomic hydrogen, which can subsequently penetrate in the bulk inducing changes of their optical properties. This provides a rather high selectivity and consequently an important advantage compared to other types of sensors. Key specifications of the sensors include their operating concentration range and limit of detection, response/recovery times, as well as their sensitivity. In addition, the sensors need to be reliable and robust, while at the same time one should be able to produce them in a simple and cost-effective manner.

Table 1 lists representative material types discussed in the previous sections, including the range of concentrations under which each sensor has been tested, reported values of their LOD, as well as their response and recovery times. Note here that LOD values are not always reported in the literature. In those cases, the lowest concentration used in each study provides an indication of the LOD, although this has to be treated with caution as the two values (i.e., lowest tested concentration and LOD) may deviate significantly from one another. In a similar manner, attention should be paid when comparing the response and recovery times of the sensors as those are determined under different conditions (most importantly at different H_2_ concentrations) by different research groups. Despite these discrepancies, the values compiled in Table 1 provide a first attempt to identify the appropriateness of each sensor for different applications. Note here that all the concentrations have been converted to mixing ratios at atmospheric pressure (express as ppb, ppm or %) to facilitate comparisons.

We should also point out that sensitivity is another important parameter that can define the appropriateness of a sensor for certain applications. For reflectance/transmittance sensors sensitivity is defined as Δp/ΔT (cf. Section 3), whereas for the plasmonic sensors as Δp/Δλ_peak_, where Δp is the difference in hydrogen partial pressure and Δλ_peak_ the resonance shift. In sensors that exhibit a linear response (i.e., the induced signal is linearly related to the concentrations of the target gas that the sensor is exposed to), the sensitivity has a fixed value. For sensors that exhibit a nonlinear response, which is the case for most of the optical H_2_ sensors, the sensitivity varies with concentration and thus it is difficult to compare among the different sensor types. Doping of both INP-based materials and thin films (e.g., with Au) can reduce the sensitivity of those sensors while the response time can be improved.

Reported concentrations at which the sensors have been tested among all the studies range from approximately 10 ppb to 32%. Similarly, LOD values for all material categories (i.e., thin films, ANP- and INP-based materials and CN) are in the range of a few tens of ppb to a few thousand ppm, as shown in Table 1, with those exhibiting lowest LOD values being the Pd-capped Ta and Hf thin films. Pd-based thin films have response times in the order of a few (for the case of Pd-caped thin films) to a few tens of s, and recovery time longer than 20 s. Those can be improved to sub-second ranges by using either ANP or INP (e.g., PdAuCu or of PdAu nanoparticles coated with PTFE and PMMA).

### 6.1. Addressing Limitations of Pd-Based of Optical H_2_ Sensing Materials

The important limitations of Pd-based H_2_ sensing materials can be addressed to a certain extent by different synthesis approaches including the use of coatings, mixed materials and by nanostructuring. The hysteresis effect typically exhibited by flat and ANP thin films can be addressed by alloying and/or controlling their thickness. In addition, small (i.e., a few nanometers) INPs can also suppress hysteresis. Polymeric coatings applied on thin films have also been shown to limit CO poisoning and enhance the chemical stability [66]. This approach can in principle be used on all the types of materials discussed here, including INP- and CN-based materials [33]. Alloy PdAuCu materials structures have also been shown to address this issue and improve the chemical stability of the sensor, suppressing the CO poisoning.

Cracking, which is typically observed in flat and ANP thin films, is oftentimes seen as a limitation because it spoils the uniformity of the materials. Nevertheless, this can be considered as advantageous as cracks increase the surface to volume ratios of the materials, thereby enhancing the overall sensor sensitivity. Cracks are by definition avoided when using INP- and ANP-based sensing materials, as the building blocks of the very materials are isolated and thus do not form large “crackable” crystal structures [41,100,101].

Overall, sensors employing INP-based materials that exhibit LSPR peak shifts upon exposure to H_2_ have been shown to be highly promising. Important advantages of INP-based materials are that they are in principle more sensitive compared to their thin-film counterparts, they show very fast response/recovery times, as well as they prevent hysteresis effect. In a similar manner, CN-based materials eliminate the hysteresis effect and at the same time to improve the response/recovery time and decrease the limit of detection of the final sensor. Despite that such materials are associated with more elaborate and precise fabrication methods, they hold great promises in making sensitive sensors [43,44].

### 6.2. Manufacturing of Materials for Optical H_2_ Sensors

An important consideration defining whether a specific sensor material can be industrially produced is the easiness, the repeatability, and the cost of manufacturing. Thin film sensors have rather established, straightforward, and easy ways of manufacturing. Common PVD techniques, such as sputtering and e-beam evaporation have been successfully used to synthesize Pd-based thin films for optical H_2_ sensors. Furthermore, sol–gel has been used as a promising fabrication technique for depositing Pd thin films.

ANP-based materials can be fabricated even easier compared to conventional thin films, using for example wet chemistry approaches [83], and atmospheric-pressure aerosol-based methods such as spark ablation and flame synthesis [2,70,71,102,103,104]. We should note here that aerosol-based fabrication methods are typically less expensive, mainly due to the lower capital cost of the setups/tools required, than PVD and lithography [44], providing promising solutions for industrial manufacturing.

INP-based materials can be fabricated by lithography, wet chemistry, as well as PVD techniques. Lithography can provide INPs in ordered array structures on the sensor substrates [33,79], while the use of PVD (followed by post-annealing treatment to confirm the fabrication of the INPs) and wet chemistry techniques can produce INPs that are randomly but homogeneously distributed on the substrates [51,55]. Such materials can in principle be produced by aerosol-based techniques, providing rather easy and cost-effective manufacturing, but this has not been tried yet to the best of our knowledge.

CN-based sensing materials can be made using more complicated tools that combine physical and chemical processes. Typically, a combination of lithography and conventional PVD techniques can be applied for the fabrication of CN-based optical H_2_ sensors, providing suitable material architectures for optical H_2_ sensing. Nevertheless, the complexity of their manufacturing processes could potentially provide limitations for industrial scaling up, preventing their adoption especially for start-up companies.

All in all, the criteria for selecting the most appropriate method for manufacturing sensing materials strongly depend on the specific demands of the final sensors. PVD methods can be used to produce conventional thin film layers, the properties of which can be tailored by alloying. To increases the surface to volume ration of thin films and thus further enhance their performance of thin films requires methods for nanostructuring, including aerosol-based or chemical solution methods. Both are rather inexpensive methods for synthesizing sensing elements, which is crucial for industrial production. Lithography-based or combination of PVD and CVD fabrication can be used to produce INP-based materials and CN architectures that can yield highly sensitive (with low-enough LOD values) and fast response sensors, but at a rather high cost.

## 7. Summary

In summary, this paper provides an overview of various Pd-based materials that can be employed in optical H_2_ sensors. The working principle in these sensors is based on the conversion of their sensing material to hydrates upon H sorption, which causes a detectable change of their optical properties, i.e., changes in the transmittance/reflectance or shifts in LSPR of the sensing material depending on the type of the sensors. Starting with a discussion on optical H_2_ sensing using thin-film materials, the paper expands on how these can be improved and how certain limitations that they exhibit can be overcome by using ANP- and INP-based materials, as well as with materials employing CNs. The last section of the paper provides a discussion where the pros and cons of each material architecture are compared, and highlights directions for further research.

## Figures and Tables

**Table 1:** Performance characteristics of Pd-based thin films and materials based on ANP, INP, or CNs for optical H_2_ gas sensors. In all cases, the sensors were operated at room temperature unless noted otherwise. For many materials (second column) the limit of detection is not directly measured, but only the lowest hydrogen concentration at which the testes were made is provided (third column). The fourth column gives the lowest detectable value (experimentally and not material limited). The fifth and sixth column provide the reported response and the recovery times of the materials at specific H_2_ concentrations and in some cases measurements that deviate from ambient (numbers in brackets). Key: NPs stands for nanoparticles, and na for not addressed/applicable.

Material Type	Sensing Material	Sensor Operating Concentration Range (ppm)	Limit of Detection (ppm)	Response Time (s) Parentheses Show the Concentration (in ppm) and Temperature	Recovery Time (s)
Thin film	Pd-capped Y thin films [63]	5–10^3^	5	10 (3 × 10^3^) 25 (10^4^)	250
	Pd-capped Mg thin films [64]	2 × 10^3^–4 × 10^4^	na	500 (4 × 10^4^)	na
	Pd-capped Ta thin films [59,67] Room temperature operation of the above sensor	10^−2^–10^5^	na 2 (room temperature)	7 (3 × 10^3^; 120 °C) <1 (room temperature)	20 ~1 (room temperature)
	Pd-capped Hf thin flms [68] PdAu thin films [37,44]	10^−2^–10^5^ ~10^−2^–10^5^	na (10^−2^) na (10^−2^)	4 (120 °C) a few seconds (28 °C)	30 (120 °C) a few tens of seconds
ANP-based	PdAu nanoparticulate material [41]	10^3^–10^6^	na (10^3^)	<10	<20
	Anisotropic nanostructured Pd thin films [78]	1–10^5^	10	0.6 (4 × 10^4^)	na
INP-based	PdAu nanodiscs on glass [45]	10^3^–10^6^	na (10^3^)	<1 (4 × 10^4^)	na
	PdNPs/fused silica [51]	10^4^ and 5 × 10^4^ (pulses)	na	2 (5 × 10^4^)	5
	PdAuCu nanoparticles [79]	10^3^–10^6^	5	0.4 (4 × 10^4^; 30 °C)	5
	PdNPs/SnO_2_ waveguide [96]	8 × 10^3^ to ~32 × 10^4^	na (5 × 10^3^)	3 (3 × 10^4^)	2
	Au@Pd NPs/quartz [97]	10^3^–4 × 10^4^	na (10^3^)	4 (4 × 10^4^)	30
	PdAuNPs@PTFE@PMMA [33]	10^2^–10^6^	10^3^	0.3 (4 × 10^4^; 30 °C)	4
CN	PdAu nanohelices [90]	10^4^–2.5 × 10^4^	na	<20 (10^3^)	<80
	*Morpho Butterfly*@Pd nanostrips [91]	10–4 × 10^4^	<10	50 (10^2^) ~20 (10^4^)	na
	PdAuNPs/optical fiber [98]	8 × 10^3^–6 × 10^4^	na (<10^4^)	2 (4 × 10^4^)	20
	PdNPs-PMMA/optical fiber [99]	2 × 10^3^–× 10^4^	35.8	5 (10^4^)	na

## Data Availability

Not applicable.
